# On and off-target effects of telomere uncapping G-quadruplex selective ligands based on pentacyclic acridinium salts

**DOI:** 10.1186/1756-9966-32-68

**Published:** 2013-09-19

**Authors:** Sara Iachettini, Malcolm FG Stevens, Mark Frigerio, Marc G Hummersone, Ian Hutchinson, Thomas P Garner, Mark S Searle, David W Wilson, Manoj Munde, Rupesh Nanjunda, Carmen D’Angelo, Pasquale Zizza, Angela Rizzo, Chiara Cingolani, Federica De Cicco, Manuela Porru, Maurizio D’Incalci, Carlo Leonetti, Annamaria Biroccio, Erica Salvati

**Affiliations:** 1 Experimental Chemotherapy Laboratory, Regina Elena National Cancer Institute, Via delle Messi D’Oro, 156 00158 Rome, Italy; 2 School of Pharmacy, University of Nottingham, Nottingham NG7 2RD, UK; 3 Pharminox Ltd, BioCity, Pennyfoot Street, Nottingham NG1 1GF, UK; 4 Center for Biomolecular Sciences, School of Chemistry, University of Nottingham, Nottingham NG7 2RD, UK; 5 Department of Chemistry, Georgia State University, P.O. Box 4098, Atlanta, Georgia 30302-4098; 6 Department of Oncology, Pharmacological Research Institute ‘Mario Negri’, Milan, Italy

**Keywords:** Telomere targeting agents, G-quadruplex, Anti-cancer therapy

## Abstract

Quadruplexes DNA are present in telomeric DNA as well as in several cancer-related gene promoters and hence affect gene expression and subsequent biological processes. The conformations of G4 provide selective recognition sites for small molecules and thus these structures have become important drug-design targets for cancer treatment.

The DNA G-quadruplex binding pentacyclic acridinium salt RHPS4 (1) has many pharmacological attributes of an ideal telomere-targeting agent but has undesirable off-target liabilities. Notably a cardiovascular effect was evident in a guinea pig model, manifested by a marked and sustained increase in QTcB interval. In accordance with this, significant interaction with the human recombinant β2 adrenergic receptor, and M1, M2 and M3 muscarinic receptors was observed, together with a high inhibition of the hERG tail current tested in a patch clamp assay.

Two related pentacyclic structures, the acetylamines (2) and (3), both show a modest interaction with β2 adrenergic receptor, and do not significatively inhibit the hERG tail current while demonstrating potent telomere on-target properties comparing closely with 1. Of the two isomers, the 2-acetyl-aminopentacycle (**2**) more closely mimics the overall biological profile of 1 and this information will be used to guide further synthetic efforts to identify novel variants of this chemotype, to maximize on-target and minimize off-target activities.

Consequently, the improvement of toxicological profile of these compounds could therefore lead to the obtainment of suitable molecules for clinical development offering new pharmacological strategies in cancer treatment.

## Background

Telomeric DNA is protected and maintained at the ends of chromosomes by the action of the enzyme telomerase. Whilst the shortening of DNA telomeres during repeated cell division is a natural part of the cellular ageing mechanism, one of the hallmarks of cancer is the expression of telomerase by cancer cells which allows them to maintain telomeric length and adopt immortal characteristics [1,2]. Telomerase requires a single-stranded DNA primer as substrate for the addition of telomeric repeats (TTAGGG) [3], his terminal telomere G-rich single stranded tract, also called G-overhang, can fold into four-stranded G-quadruplex (G4) structures consisting of G-tetrads coordinated around a monovalent cation [4,5]. G4 stabilization, deny access of telomerase to its substrate, representing a valid tool for telomerase targeted approach in cancer therapy [6]. Nevertheless, for direct telomerase inhibition, a time-dependent response is observed, related to the basal length of the telomeres, due to the slow attrition of telomeres experienced after each cell division, thus limiting the efficacy of agents designed to inhibit telomerase alone [7-9]. The extremely rapid and potent cytotoxic effect triggered by G4 ligands interacting with telomeric DNA sequences (‘Telomere Targeting Agents’: TTAs) is explained by a dual mechanism of action. On one hand the inhibition of telomerase, and, on the other hand, disruption of the shelterin complex, a nulcleo-protein complex which stabilises and protects the ends of chromosomes from being recognized as double-strand breaks [7,8]. The presence of G4 structures has been recently showed in non telomeric regions, as already hypothesized on the base of predictive studies [10]. In particular, G4 forming regions were already found in the promoter of several cancer related genes (c-myc, bcl2, hif1, hTERT), and for some of those genes, a transcriptional inhibitory function was attributed to these structures. Consequently, G4 targeting molecules could have additional extra-telomeric features, which could improve their potential as anti-cancer agents.

**Figure 1:**
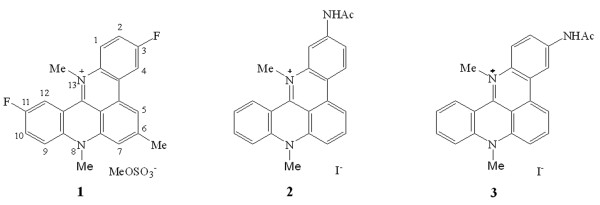
Structures of quinoacridinium salt RHPS4 (1) and related chemotypes (2 and 3).

The pentacyclic quinoacridinium salt RHPS4 (1: Figure 1) has many of the attributes of an ideal TTA. We have shown previously by NMR studies that the agent stacks above and below the G3 core of a (TTAGGGT)_4_ parallel-stranded quadruplex; [11] it binds with high efficiency to the *h*-Tel DNA sequence as measured by surface plasmon resonance [11], circular dichroism and ESI-MS [12,13]; it is an active inhibitor of telomerase (IC_50_ 0.33 μM) as revealed in a Trap assay [14] and disrupts the shelterin complex of the telomere with the liberation of the POT-1 protein [15]. The compound shows high efficacy in *in-vitro* tumor cell growth inhibition assays [14-16] and is particularly effective against human tumor xenografts in combination with taxol, [17] irinotecan [18] and, spectacularly so, in a triple combination with irinotecan and a PARP-1 inhibitor [19]. In addition, the compound has some desirable chemical and pharmaceutical properties such as ease of synthesis by a two-step route [20], high solubility, stability, and predicted freedom from metabolic liabilities [21]. However, in this paper we report that the prototypic quinoacridinium salt 1 also exhibits some undesirable off-target effects, but that these effects can be ameliorated to some extent in related non-fluorinated compounds 2 and 3 without compromising on-target properties. These physico-chemical and pharmacological studies offer hope that a suitable clinical candidate might yet emerge based on this pentacyclic chemotype.

## Methods

### Chemistry

3,11-Difluoro-6,8,13-trimethyl-8*H*-quino[4,3,2-*kl*]acridinium metho-sulfate 1 was prepared from 6-fluoro-1,2-dimethylquinolinium methosulfate **7** as described [17]. 2-Acetylamino- (2) and 3-acetylamino-8,13-dimethyl-8*H*-quino[4,3,2-*kl*]-acridinium iodide (3) were prepared according to published methods [20].

### 13-Ethyl-3,11-difluoro-6,8-dimethyl-8*H*-quino[4,3,2-*kl*]acridinium trifluoromethosulfate (8)

Ethyl trifloromethosulfate (1 mL) was added to a solution of 3,11-difluoro-6,8-dimethyl-8*H*-quino[4,3,2-*kl*]acridine (6; 0.05 g, 0.15 mmol) in CHCl_3_ (2 mL) under nitrogen. The mixture was heated at 140°C in a sealed tube for 3 days, cooled and solvent evaporated. The residue was purified by column chromatography on silica gel (5% MeOH/DCM) to leave the salt (8) as a bright red solid (20%), mp >250°C (decomp.); IR (ν_max_) 1620, 1583, 1533, 1475, 1429, 1255, 1028 cm^-1^; ^1^H NMR (DMSO-*d*_6_) δ 8.58 (1H, dd, *J* = 10.0, 2.9 Hz), 8.43 (1H, s), 8.26 (2H, m), 8.21 (1H, dd, *J* = 9.4, 4.9), 8.04 (1H, m), 8.01 (1H, s), 7.78 (1H, m), 5.12 (2H, q, *J* = 6.8 Hz, N-CH_2_), 3.17 (3H, d, *J* = 5.1 Hz), 2.78 (3H, s, N-CH_3_), 1.15 (3H, t, *J* = 6.8 Hz, N-CH_2_*CH*_*3*_); *m/z* 361.1 (M^+^).

### Cardiovascular effects of anaesthetised Guinea pig

After anaesthesia with approximately 40 to 60 mg/kg (i.p.) sodium pentobarbitone, a jugular vein was cannulated for administration of the vehicle or test substance. Arterial blood pressure (systolic, diastolic and mean) was measured via a catheter inserted into the carotid artery, heart rate was derived electronically from the pressure waveform and a sample of arterial blood determined blood gases (PO_2_ and PCO_2_), O_2_ saturation, standard bicarbonate (HCO_3_), pH and base excess before the start of the experiment. Electrocardiogram (ECG) limb electrodes recorded the standard lead II configuration and QTcB interval (calculated as QTcB = QT/(√RR)). The animal was allowed to stabilise after completion of the surgical preparation for a period of at least 15 min. Then, after a further 10 min period of continuous recording of ECG and haemodynamic variables, the test substance or vehicle was administered intravenously as 3 iv infusions with each administration separated by 60 min.

### Receptor inhibition

For hERG study, HEK293 cells were cultured (1–7 days) in DMEM/GlutaMax-1 + 10% FBS and were plated on collagen-coated dishes (about 2×10^4^ cells/dish). The cell was held at -80 mV. A 50-millisecond pulse to -40 mV was delivered to measure the leaking currents, which were subtracted from the tail currents online. Then the cell was depolarized to +20 mV for 2 seconds, followed by a second pulse to -40 mV for 1 second to reveal the tail currents. This paradigm was delivered once every 5 seconds to monitor the current amplitude. After the current amplitude stabilized, the test compound was delivered to the extracellular medium by a rapid solution changer perfusion system. During perfusion, the cell was repetitively stimulated with the protocol described above, and the current amplitude was continuously monitored. Data were acquired and analyzed by using pClamp (Axon Instruments), and Excel (Microsoft), and are reported as mean and individual values. The degree of inhibition (%) was obtained by measuring the tail current amplitude before and after drug superfusion (the difference current was normalized to control and multiplied by 100 to obtain the percent of inhibition). Concentration (log) response curves were fitted to a logistic equation (three parameters assuming complete block of the current at very high test compound concentrations) to generate estimates of the 50% inhibitory concentration (IC_50_). The concentration-response relationship of each compound was constructed from the percentage reductions of current amplitude by sequential concentrations. β2-adrenergic receptor CHO expressing cells were used for the receptor inhibition assay as described [22]. The results are expressed as a percent of inhibition of control specific binding (100 - (measured specific binding/control specific binding) × 100)) obtained in the presence of the test compounds. The specific ligand binding to the receptors is defined as the difference between the total binding and the nonspecific binding determined in the presence of an excess of unlabelled ligand. All the in-vivo experiments were carried out at the Regina Elena Cancer Institute. All procedures involving animals and care were performed in compliance with our institutional animal care guidelines and with international directives (directive 2010/63/EU of the European parliament and of the council; Guide for the Care and Use of Laboratory Animals, United States National Research Council, 2011).

### Biosensor-surface plasmon resonance (SPR) studies

Oligonucleotides 5′-biotin-d[AG_3_(T_2_AG_3_)_3_] quadruplex and 5′-biotin-CGA_3_T_3_C(CT)_2_GA_3_T_3_CG were purchased from Midland Certified Reagent Company (Midland, TX). Purification of DNA, preparation of solutions, collection of data, and analysis of results were conducted according to methods adopted in an earlier study [11].

### CD spectroscopy

CD spectra were recorded on an Applied Photophysics Pi-Star-180 spectrophotometer (Applied Photophysics Ltd, Surrey, UK). The optical system was configured with a 75 W Xe lamp, circular light polarizer and end-mounted photomultiplier. The instrument had previously been calibrated with (D)-camphorsulfonic acid. Temperature was regulated using a Neslab RTE-300 circulating programmable water bath (Neslab Inc). CD spectra were recorded at 298 K in a 10 mm path length cell over a wavelength range of 215–345 nm in steps of either 1 0r 2 nm, with 3 nm entrance/exit slit widths: the number of counts was set to 10,000 with adaptive sampling set to 500,000. The spectra were corrected by subtracting the spectrum of the same buffer solution of 100 mM potassium chloride and 10 mM potassium phosphate at pH 7.0. Annealing and melting profiles were recorded using a thermoelectric temperature controller (Melcor) on 4 *μ*M DNA samples with and without 3.5 mol.equiv. of ligands using 0.5 K temperature increments and a cooling or heating rate of 0.2 K/min over the temperature range 298-368 K.

### Cells and culture conditions

BJ fibroblasts expressing hTERT (BJ-hTERT) or hTERT and SV40 early region (BJ-EHLT), were obtained as previously reported [15]. Cells were grown in Dulbecco Modified Eagle Medium (D-MEM, Invitrogen Carlsbad, CA, USA) supplemented with 10% fetal calf serum, 2 mM L-glutamin and antibiotics.

### Proliferation assay

5 × 10^4^ cells were seeded in 60-mm Petri plates (Nunc, MasciaBrunelli, Milano, Italy) and 24 h after plating, 0.5 μM of freshly dissolved compound was added to the culture medium. Cell counts (Coulter Counter, Kontron Instruments, Milano, Italy) and viability (trypan blue dye exclusion) were determined daily, from day 2 to day 8 of culture.

### Immunofluorescence

Cells were fixed in 2% formaldehyde and permeabilized in 0.25% Triton X100 in PBS for 5 min at room temperature. For immunolabeling, cells were incubated with primary antibody, then washed in PBS and incubated with the secondary antibodies. The following primary antibodies were used: pAb and mAb anti-TRF1 (Abcam Ltd.; Cambridge UK); mAb (Upstate, Lake Placid, NY) and pAb anti-γH2AX (Abcam). The following secondary antibody were used: TRITC conjugated Goat anti Rabbit, FITC conjugated Goat anti Mouse (Jackson ImmunoResearch Europe Ltd., Suffolk, UK). Fluorescence signals were recorded by using a Leica DMIRE2 microscope equipped with a Leica DFC 350FX camera and elaborated by a Leica FW4000 deconvolution software (Leica, Solms, Germany). This system permits to focus single planes inside the cell generating 3D high-resolution images. For quantitative analysis of γH2AX positivity, 200 cells on triplicate slices were scored. For TIF’s analysis, in each nucleus a single plane was analyzed and at least 50 nuclei per sample were scored.

### Fluorescence in situ hybridization (FISH)

For metaphase chromosome preparation cells were treated with demecolcine (Sigma, Milan, Italy) 0.1 mg/ml for 4 h and then harvested and washed in 75 mM KCl for 5 min at 37°C. After centrifugation cells were fixed in MeOH/acetic acid 3:1 overnight and then spread on slides. Hybridization with rhodamine-coupled PNA was performed as described [23]. For each sample 20 metaphases per slice on triplicate were scored. Images of the metaphases were captured with a 100 × objective.

### Chromatin immunoprecipitaion assay (ChIP)

BJ-EHLT fibroblasts were treated for 24 hrs with 0.5 μM of the compound. ChIP assay was performed as previously described [24]. The following antibodies were used: pAb anti-TRF1 (Santa Cruz Biotechnology, Santa Cruz, Ca); mAb anti-TRF2 (Imgenex, San Diego, CA); pAb anti-POT1 (Abcam). mAb anti-β-actin (Sigma) was used as negative control of the ChIP assay.

## Results and discussion

### Synthesis of quino [4,3,2-*kl*] acridinium salts

**Figure 2:**
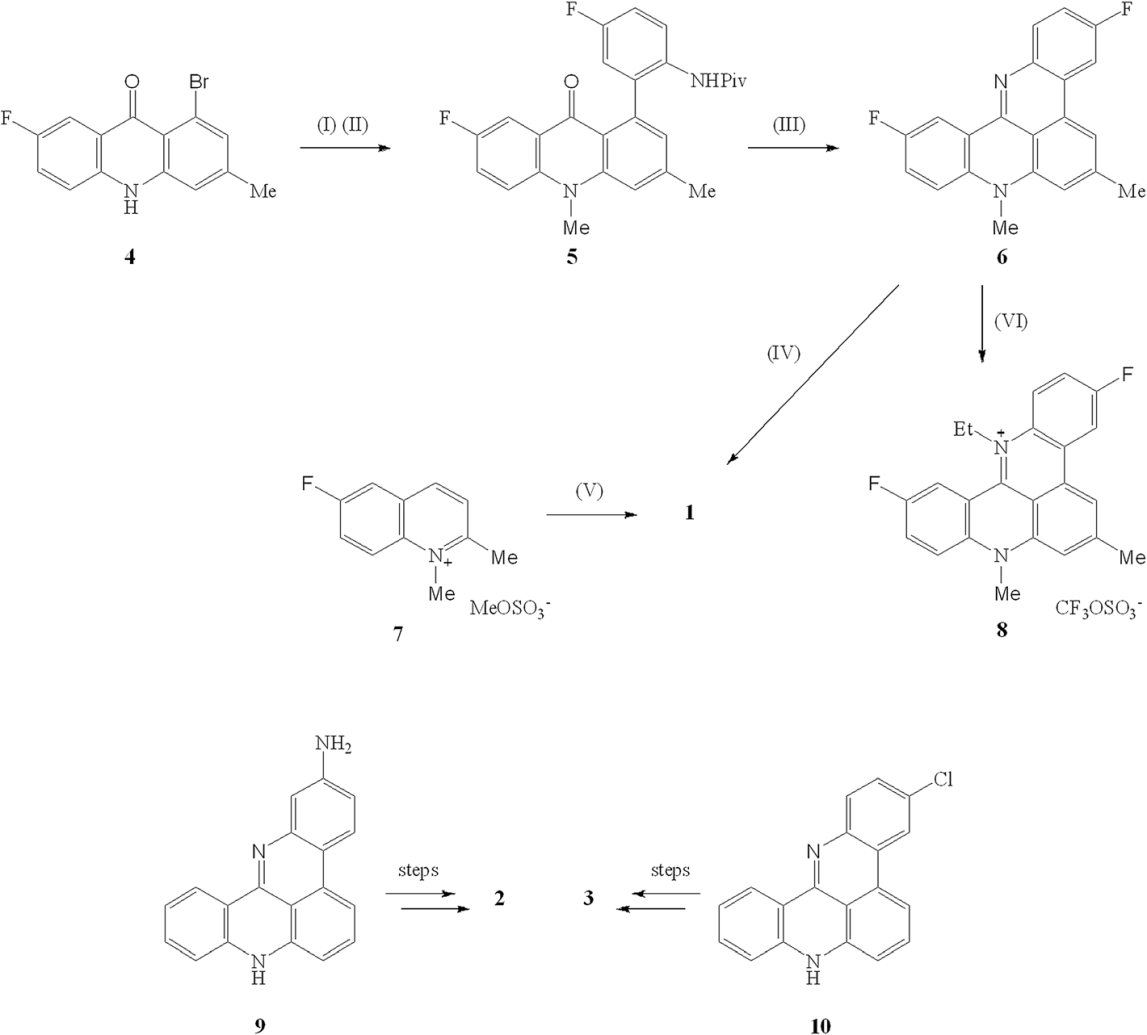
**(I) NaH in DMF, Me**_**2**_**SO**_**4**_**; (II) 5**-**fluoro**-**2**-**pivalamidophenylboronic acid**, **Pd**(**PPh**_**3**_)_**4**_, **NaHCO**_**3**_, **aq. DME, 80°C; (III) EtOH, 5 M HCl; (IV) Me**_**2**_**SO**_**4 **_**in MeNO**_**2**_**, reflux; (V) Et**_**3**_**N in nitrobenzene, 120°C, 24 h; (VI) EtOSO**_**2**_**CF**_**3**_**, CHCl**_**3**_**, 140°C, 72 h.**

We have previously reported two routes to the pentacyclic acridinium salt 1. The less efficient involved construction of the 1-bromo-7-fluoro-3-methylacridone 4 which was processed to the intermediate *N*-methylacridone 5 where the pivaloyl-protected fluoroaniline fragment was attached by a Suzuki coupling.^18^ Deprotection and cyclization of 5 in EtOH–5 M HCl yielded the pentacycle 6 in 65% yield (Figure 2). Methylation of 5 with dimethyl sulphate in refluxing nitromethane furnished the dark red pentacyclic salt 1 (48%). However the overall yield of 1 from suitable precursors to 4 was disappointingly low at < 10% [20]. A more practicable route for the large-scale synthesis of salt 1 involved the multi-step, but ‘one-pot’, conversion of the *N-*methylquinolinium methosulfate salt **7** to 1 with triethylamine in nitrobenzene at 120°C in an optimized 33% yield [21]. This surprising process was adapted from a synthesis of salts related to 1 by Ozczapowicz and colleagues in 1988 [21].

Efforts to prepare higher alkyl homologues of 1 were only partially successful presumably because access by larger alkylating moieties at N-13 of pentacycle 6 were impeded by hydrogen atoms at positions 1 and 12 (for numbering system see Figure 1): thus whereas the *N*-ethyl quaternary salt 8 (20%) could be prepared with difficulty by heating 6 and ethyl trifluoromethane sulfonate in chloroform under nitrogen at 140°C in a sealed tube for 3 days, it was not possible to prepare *n*-propyl or *i*-propyl homologues of 6 under a range of forcing conditions. The isomeric *N*-acetyl compounds 2 and 3 were prepared starting from the 2-aminoquinoacridine 9 or 3-chloroquinoacridine 10, respectively, in several steps according to our previously published work [25].

### Toxicity of quinoacridinium salt 1

Initial in vivo evaluation of 1, in human tumor xenografted nude mice, did not indicate any toxicity at efficacious doses, as no toxic deaths or body weight loss was observed during or after treatment. Furthermore, histological analysis, done at the end of treatment with 1, revealed no evidence of lesions or morphological alterations in the organs and tissues examined. Nevertheless, just after 1 administration, a marked but reversible hypotension was observable, accompanied by a heart rate and cardiac output decrease in the treated compared to the control mice [18].

**Figure 3:**
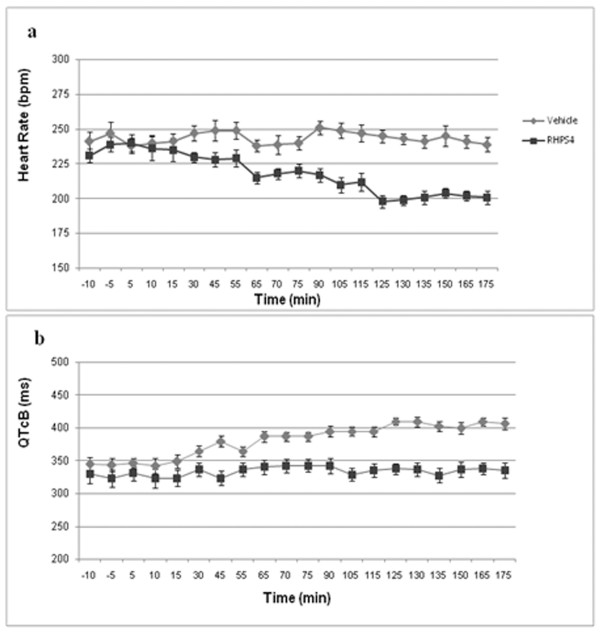
**Cardiovascular effects of 1.** Effect of 1 on Heart Rate **(a)** and QTcB Interval **(b)** in the Anaesthetized Guinea Pig Following Escalating Intravenous Doses of 0.25 mg/kg, 0.5 mg/kg and 1.0 mg/kg (Cumulative Dose: 1.75 mg/kg). Sequential doses of 0.25 mg/kg, 0.5 mg/kg and 1.0 mg/kg to each of three guinea pigs. Time interval between doses: 60 minutes. Plots represent mean data from three animals.

Several limitations exist in the use of mouse and rat models for the study of cardiotoxic effects in pharmacology, which regard differences in myocardial function compared to the human heart [26]. To better address the question of cardiac toxicity of 1, more detailed study was conducted in anaesthetised guinea pigs, where application of an infusion pump allowed for a constant rate of drug delivery over a period of up to 1 hour; this method also allowed for repeated administration of 1, with dose escalation, in the same animal over a 3 hour period. At escalating doses of 0.25, 0.5 and 1 mg/kg, each administered to the same guinea pig (n = 3) over a 5 minute period, and with each dose separated by a period of 1 hour (cumulative dose 1.75 mg/kg), there was a dose-related decrease in heart rate during the course of the experiment (Figure 3a). Significantly, a marked and sustained decrease in the QTcB interval (QT interval corrected for heart rate) was observed at all doses (Figure 3b).

To investigate the molecular bases of cardio toxicity, the agent was tested for its potential to interact with a panel of 54 pharmacological receptors, the majority being human recombinant receptors (Cerep ExpresSProfile screen) (http://www.cerep.fr). At a concentration of 1 μM significant interaction (% inhibition of ligand binding >95%) was demonstrated with the β2 adrenergic receptor (Table 1), and M1, M2 and M3 muscarinic receptors (data not shown); of more concern, compound 1 was also classified as a highly potent inhibitor of the hERG (human Ether-a-go-go Related Gene) tail current when tested in a conventional patch clamp assay (100% inhibiton at 10 μM, Table 1), which can be predictive of possible cardiovascular complications in clinical development [27].

**Table 1:** On and off target profile of pentacyclic acridinium salts 1, 2 and 3

**Compound**	**Off-target effects: cardiac receptor inhibition**		**On-target effects: ligand-quadruplex interaction**				
	**hERG % inhib. (10 μM)**	**B2 adrenergic % inhib. (10 μM)**	**Surface plasmon resonance**^**a **^**(K x 10**^**7**^ **M**^**-1**^**)**			**CD study thermal stability**^**d**^	
			**Quadruplex (Q) DNA**^**b**^	**Duplex (D) DNA**^**c**^	**Ratio Q/D**	**T**_**m**_**/°C**	**∆T**_**m**_^**e**^
**1**	100	100	0.83	0.06	13.8	86 ± 3	16
**2**	42.5	24	1.5	0.04	37.5	89 ± 3	19
**3**	21	29	0.8	0.07	11.4	85 ± 3	15

^a^Buffer solution: 10 mM HEPES, 200 mM KCl, 3 mM EDTA and 0.01% P20 surfactant with the final pH adjusted to 7.4.

^b^Human telomeric sequence 5′-d[AGGG(TTAGGG)_3_]-3′.

^c^5′-d[CGA_3_T_3_C(CT)_2_GA_3_T_3_CG]-3′ hairpin sequence.

^d^Thermal stability data for *h*-Tel (anti-parallel) determined by CD in the absence and presence of compounds.

^e^T_m_ for unaligned *h*-Tel = 70 ± 3.

### Ligand redesign to minimize off target effects

The potent hERG inhibition compromised the acceptability of 1 as a clinical candidate, despite this agent having many of the attributes of an ideal pharmaceutical [28]. Two strategies have been adopted in an attempt to minimize the hERG interaction: (i) sterically masking the (delocalized) positive charge on the acridinium cation by increasing the size of the substituent at position 13 as in compound 8; and (ii) evaluating compounds 2 and 3 as prototypes of two series of isomeric pentacyclic acridinium salts of the same chemotype as 1.

hERG tail current inhibition was used as a marker of potential off-target liabilities. The prototypic agent 1 potently inhibited hERG by 100% at 10 μM (IC_50_ 0.2 μM) (Table 1); inhibition of hERG was reduced to 43% at 10 *μ*M (IC_50_ 3.7 μM) in the 2-acetylaminoquinoacridinium iodide 2 and to 18% by 13-ethyl homologue 8, while the least potent hERG inhibitor (IC_50_ 18 μM) was the 3-acetylamino isomer 3, a 90-fold improvement over 1. The marked improvement of 8 over 1, was paralled by a >10-fold reduction in the on-target effect against the *h*-Tel DNA sequence as measured by surface plasmon resonance (see below) suggesting that increasing the size of the onium head was not a fruitful developmental approach, for these reason the compound 8 was excluded from further studies.

The interaction with β2-adrenergic receptor was determined by a binding assay of 1, 2 and 3 to the transgenic β2-adrenegic receptor expressed on the surface of CHO cells. Inhibition of receptor was reported as inhibition of control specific binding (100 - (measured specific binding/control specific binding) × 100) obtained in the presence of the test compounds. A decay of 75% and 70% of receptor inhibition is observed comparing 1 to 2 and 3 compounds respectively (Table 1). These results indicate that potential toxicities in this chemotype, as predicted by hERG and β2-adrenergic receptor interactions, can be addressed by suitable molecular modification.

### On target-effects: ligand-quadruplex interactions

**Figure 4:**
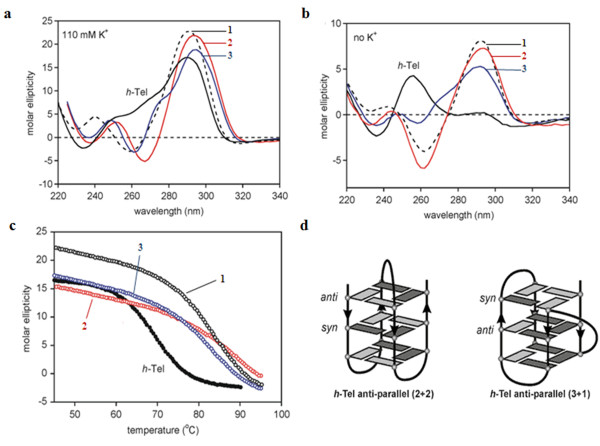
**Ligand-quadruplex interactions. a**: CD spectra of ligands 1 (black hashed), 2 (red) and 3 (blue) at 4 μM in 110 mM of K^+^ at pH 7.0 using the *h*-Tel sequence 5′-d[AGGG(TTAGGG)_3_]-3′ are compared to the ligand free *h*-Tel (black) spectra. **b**: The ability of ligands 1, 2 and 3 to induce an anti-parallel conformation in the absence of K^+^ ions. **c**: The quadruplex stabilising effects of ligands 1, 2 and 3 was carried out by monitoring the *h*-Tel thermal unfolding at 290 nm. **d**: Schematic representation of the intramolecular folded structure of the *h*-Tel sequence 5′-d[AGGG(TTAGGG)_3_]-3′ anti-parallel (2 + 2) basket form and anti-parallel (3 + 1) hybrid form. The arrows indicate strand direction from 5′ to 3′.

The Surface Plasmon Resonance (SPR) technique is a powerful tool to compare binding affinities for G-quadruplex binding agents [11,29]. When the *h*-Tel DNA sequence comprising 5′-d[AGGG(TTAGGG)_3_]-3′ is immobilised on a sensor chip surface, binding of drug elicits a refractive index change at the surface, and hence the refractive light angle at which SPR is observed. SPR can also be used to analyze the binding of compounds to an alternating hairpin duplex sequence thus allowing accurate comparisons of the relative affinities of compounds for DNA G-quadruplex and DNA duplex structures. We have reported earlier preliminary equilibrium binding affinities of compounds 1–3 indicating that these compounds bind more strongly to the *h*-Tel quadruplex sequence; in the earlier case a duplex sequence comprising an alternating GC eight base pair hairpin was used [11]. In the present work these measurements were repeated comparing ligand binding to the same *h*-Tel sequence but with a different duplex sequence 5′-d[CGA_3_T_3_C(CT)_2_GA_3_T_3_CG]-3′ as comparator. As indicated in Table 1 compound 1 binds potently to the *h*-Tel (Q) quadruplex (K = 0.83 × 10^7^ M^-1^) and an order of magnitude less effectively to the duplex (D) sequence, with a Q/D ratio of 13.8. Very similar results were observed with the 3-acetylamino derivative 3 whereas the 2-acetylamino isomer 2 was both the most potent binder to *h*-Tel quadruplex and the weakest binder to duplex DNA, giving a more favourable Q/D ratio of 37.5. The 13-ethyl homologue 8 was a much weaker binder than the close variant (1) to *h*-Tel quadruplex (K = 0.06 × 10^7^ M^-1^) suggesting that steric perturbations imposed by the larger ethyl group compromised stacking interactions within the quadruplex structure (see Additional file 1 for sensorgrams). Quadruplex-ligand interaction was also explored by Circular Dichroism (CD). Far-UV CD spectra were collected on 4 μM samples of *h*-Tel in 110 mM K^+^ solution (100 mM KCl and 10 mM potassium phosphate buffer) at pH 7.0 and 298 K. The CD spectrum was characterised by a strong band at 295 nm with additional broad positive bands at 250 and 270 nm [12,13]. The ability of 1 and the two *N*-acetyl compounds to bind to these folded structures in K^+^ solution is shown in Figure 4a. All three ligands induce stronger ellipticity at 290 nm and a negative band at around 260 nm. These pronounced spectroscopic changes are consistent with the ligands perturbing the equilibrium in favour of the basket-type (2 + 2) anti-parallel quadruplex structure, which is the predominant species identified for the same sequence in Na^+^ solution (Figure 4d) [30-33]. The ligand-induced interconversion of structures is consistent with stabilisation of the complex through ligand contacts specific to the hydrophobic pocket created by the diagonal TTA loop over the terminal G-tetrad [12,13].

The ability of the three ligands to induce structure in the single stranded *h*-Tel sequence in aqueous solution in the absence of significant concentrations of K^+^ ions was also investigated. The unfolded *h*-Tel sequence at 298 K gives a low intensity positive band in the CD spectrum at 265 nm (Figure 4b). However, in the presence of 3.5 molar equivalents of ligand, emergence of the characteristic band at 290 nm was observed, consistent with the ligand-induced formation of the anti-parallel structures evident in the K^+^ buffered solution. Thus, under both sets of conditions (with and without stabilising K^+^ ions), evidence is adduced for ligand selectivity for the anti-parallel quadruplex structure [12,13].

This analysis was extended to examine the effects of ligand binding on thermal stability by measuring the unfolding curves at 290 nm of the complexes formed in K^+^ solution, corresponding to the CD spectra shown in Figure 4a. Monitoring the thermal unfolding transition for *h*-Tel produces a sigmoidal unfolding curve with a transition mid-point T_m_ value of 72 ± 3°C (Figure 4c). All three ligands show significant effects in enhancing the stability of the quadruplex by shifting the T_m_ values to higher temperatures (∆T_m_ ~ 15-19°C compared to *h*-Tel without bound ligands) (Table 1).

### Biological effects of quinoacridinum salts

**Figure 5:**
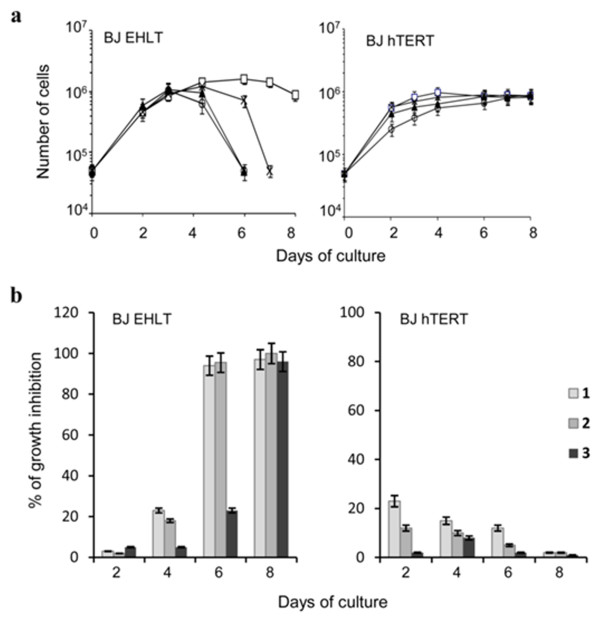
**Anti-proliferative effect on normal and transformed fibroblasts. a**: In vitro growth curves of human transformed (BJ-EHLT) and normal telomerised (BJ-hTERT) fibroblasts exposed to 0.5 μM of 1 (○), 2 (▲) and 3 (×) or vehicle (□). The number of viable cells was determined daily. Three independent experiments were evaluated. Error bars indicate SD. **b**: Histograms show the percentage of growth inhibition of BJ-EHLT and BJ-hTERT cells treated with 0.5 μM of each compound versus untreated samples at the indicated times.

To ascertain if the compounds 2 and 3 maintained the same biological and molecular features of the previously described 1, we firstly evaluated their effect on cell proliferation in a panel of different histotype tumor cell lines, showing that both compounds maintained an anti-proliferative effect in several human cancer cell lines (Additional file 1). Selectivity for transformed vs normal cells was assessed in the hTERT immortalized BJ human fibroblasts infected or not with the Large T antigen of SV40. Figure 5a and b shows the growth curves of untreated and drug-treated cells, analyzed from day 2 to 8 of culture by using 0.5 μM concentration of each compound, a dose causing cell death when cells are chronically exposed to the lead compound 1. A time-dependent decrease of cell proliferation was observed in SV40 transformed (BJ-EHLT) cells treated with the ligands reaching the maximum effect at day 6 (for the compounds 1 and 2) or seven (compound 3). Interestingly, as already described for 1, the compounds 2 and 3 did not induce inhibition of cell proliferation in normal telomerized fibroblasts, which were unaffected by the treatment (Figure 5a and b). Even if the mechanism(s) of selectivity towards transformed cells were not identified yet, our results indicate that the new-generated agents 2 and 3, similarly to the lead compound, preferentially limit the growth of cancer cells.

**Figure 6:**
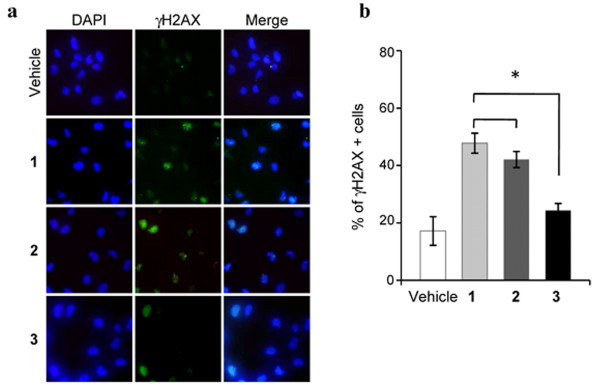
**Activation of DNA damage response.** Human transformed BJ-EHLT fibroblasts were treated with 0.5 μM of 1, 2 and 3 for 24 hrs, then fixed and processed for IF analysis with anti-γH2AX antibody, and counterstained with DAPI to mark nuclei. **a**: Representative images of IF at 63× magnification. **b**: Histograms shows the percentage of γH2AX-positive cells scored by immunofluorescence analysis (*P < 0.05).

Consequently, the ability of the new-generated G-quadruplex ligands 2 and 3 to cause telomere uncapping has been investigated. To this aim, a two-steps analysis was performed to establish, in a first case, if the compounds are able to induce DNA damage and, secondly, if the DNA damage is localized at the telomeres. Immunofluorescence analysis performed to evaluate the phosphorylation of H2AX, a hallmark of DNA double-strand break, showed that all the compounds activated a DNA damage response pathway (Figure 6a). However, the quantitative analysis revealed that the compound 2 induced a percentage of cells positive for γH2AX quite similar to compound 1, while, consistently with the above reported data on cell proliferation (Figure 5), 3 is less potent than the lead compound (*P < 0.05), in activating the damage response pathway.

**Figure 7:**
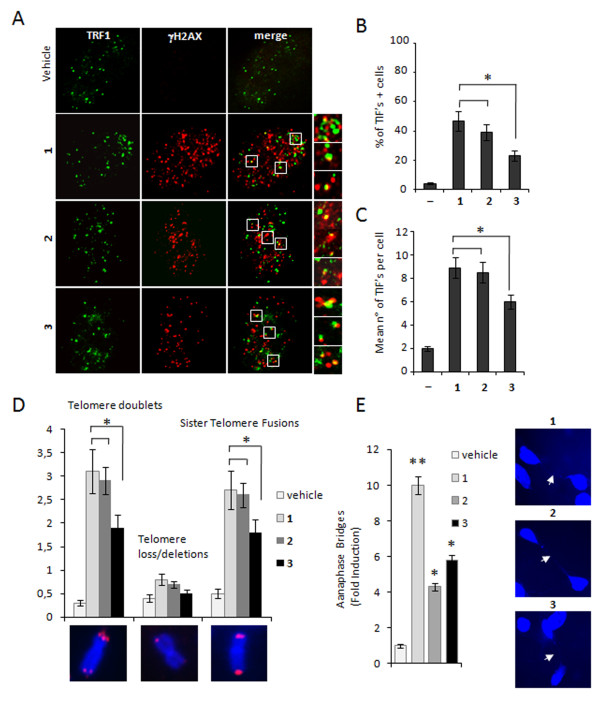
**Induction of telomere damage and aberrations.** Cells untreated or treated with 1, 2 and 3 for 24 hrs were fixed and processed for IF analysis against TRF1 and γH2AX. **a**: Representative images of IF were acquired with a Leica deconvolution microscope (magnification 100×). Enlarged views (2.5×) of treated merged images are reported. Histograms represent the Percentage of TIFs-positive cells. **b**: and average number of TIFs per nucleus. **c**: in the untreated (vehicle) and treated cells with the indicated compounds. Cells with four or more γH2AX/TRF1 foci were scored as TIF-positive. **d**: BJ-EHLT fibroblasts treated with 1, 2 or 3 for 72 hrs at 0.5 μM were blocked in metaphase with colcemide and processed for FISH with a telomere specific probe. The average number of specific telomeric aberrations in each sample is reported in the histograms. In the lower panels, representative images of telomere aberrations are shown at 100× magnification. **e**: BJ-EHLT treated with 1, 2 or 3 for 24 hrs at 0.5 μM were fixed and stained with DAPI. Anaphase bridges were scored by counting 400 nuclei per sample in triplicate. Histograms show the fold induction in treated versus untreated samples. Right panels show representative images of anaphase bridges in the treated samples at 63× magnification. Histograms show the mean of three independent experiments. Error bars indicate ± SD. (*P < 0.05).

These results encouraged us to undertake further studies aimed to investigate the telomere specific effects of the ligands, analyzing whether γH2AX was phosphorylated in response to dysfunctional telomeres. Deconvolution microscopy showed that, similarly to 1, some of the damaged foci induced by 2 and 3 co-localized with TRF1, an effective marker for interphase telomeres, forming so-called Telomere dysfunction Induced Foci (TIFs) [34] (Figure 7a). Quantitative analysis revealed that treatment with 2 increased the percentage of cells with more than four γH2AX/TRF1 co-localizations (indicated as TIF-positive cells), at comparable levels with respect to 1, while 3 had a significant but less pronounced effect. Consistently with these results, while 1 and 2 induced a superimposable number of TIFs per nucleus (ca. eight) the mean of telomere foci induced by 3 was reduced to six (Figure 7b, c).

To directly evaluate telomere damage elicited by the different ligands, the telomere status of drug-treated BJ-EHLT was analysed by a fluorescence in situ hybridization on metaphase spreads with a telomere specific fluorescent probe. The cytogenetic analysis revealed that all the compounds induced a significant increase of frequency of telomere doublets (characterized by a double telomere signal at chromosome ends) and sister telomere fusions (in which two sister chromatids telomeric signals are fused into one single spot), while other telomere aberrations (telomere losses and/or deletions) were not found. However, again telomere aberrations induced by 2 are quantitatively similar to the lead compound, while a lower effect was observed upon treatment with 3. As a result of chromosome ends fusion consequent to telomere damage, chromatin bridges are occasionally observed between daughter cells after mitosis (also called anaphase bridges). In 1 treated BJ-EHLT, anaphase bridges frequency in a cycling population was ten-fold increased. With a minor extent 2 and 3 were both able to induce anaphase bridges when administered at the same dose, closely comparing the effects of the lead compound (Figure 7e).

**Figure 8:**
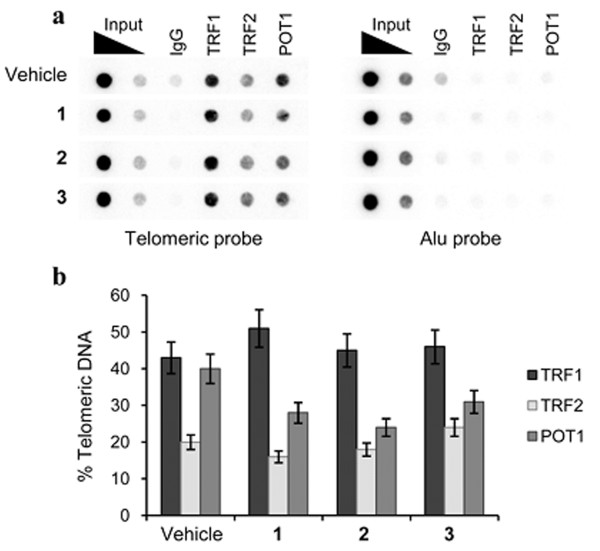
**Expression of TRF1, TRF2 and POT1 at the telomere level: ChIP experiments on BJ-EHLT fibroblasts incubated with 0.5 μM of 1, 2 or 3 for 24 hrs.** Precipitations were performed with antibodies against TRF1, TRF2 and POT1. The total DNA (input) represents 10% of genomic DNA. **a**: A representative ChIp experiment is shown. **b**: The histogram shows the densitometric evaluation of three independent experiments, with error bars indicating ± SD.

Finally, the effect of the G-quadruplex ligands on telomere capping has been investigated. Specifically, the activity of 2 and 3, in comparison to 1, was analysed on the localization of TRF1, TRF2, and POT1, three telomeric proteins that induce telomere dysfunction and evoke DNA damage signaling when their levels are reduced at telomeres. ChIP assay showed that all the compounds delocalized POT1 from telomeres, (Figure 8a and b), while TRF1 and TRF2 remained associated with the telomeres upon treatment.

This response is typical of the telomere deprotection occurring during cellular senescence or upon the loss of telomeric proteins [34-40]. The ability of G-quadruplex ligands to uncap telomeres and to possess anti-tumor activity has been already described for other agents, [41-45] reinforcing the notion that these agents can act as inhibitors of a telomere-related process and therefore the rationale for the development of this class of inhibitors as anti-tumor agents must be found elsewhere other than in higher telomerase expression in cancer cells.

Taken collectively our results clearly demonstrate that compounds 2 (but less efficiently 3) rapidly disrupt telomere architecture of cells, by delocalizing the telomeric protein POT1, resulting in a potent DNA damage response characterized by the formation of several telomeric foci.

Furthermore, it is apparent that the 2-substitued quinoacridinium salt 2 more closely mimics the overall pharmaceutical profile of the prototypic compound 1 than the regioisomer 3. Our recent synthetic work has therefore focused on the 2-substituted series and our efforts to maximize on-target and minimize off-target properties will be reported separately.

## Conclusions

Molecular modification of quinoacridinum salts 1 have shown to reduce undesired cardiotoxic effects while maintaining the on-target features as telomere targeting agents. This findings provide a strong rational for development of this class of compounds as tools for a G-quadruplex targeted anti-cancer therapy.

## Competing interests

I declare that the published research was conducted in the absence of any commercial or financial relationships that could be construed as a potential conflict of interest. Costs of experiments described within this manuscript were funded by Pharminox Ltd. The costs of the biological experiments were funded by Italian Association for Cancer Research (AIRC # 11567). Dr. S. Iachettini is recipient of a fellowship from the Italian Fundation for Cancer Research (FIRC). Dr. A. Rizzo is recipient of a fellowship from the Veronesi Foundation.

## Authors’ contributions

MFGS, MD’I, MSS and AB designed research and wrote paper; CD’A, SI, AR, CC, FDC performed biological experiments; PZ, and ES collected and analyzed biological data; MF, MGH, IH, TPG, WDW, MM, RN performed chemical experiments and collected/analyzed data. All authors read and approved the final manuscript.

## Supplementary Material

Additional file 1 Cytotoxicity of 2 and 3 and SPR sensorgrams. Click here for file
