# Supplementary material for: On and off-target effects of telomere uncapping G-quadruplex selective ligands based on pentacyclic acridinium salts
Source: J Exp Clin Cancer Res. 2013 Sep 19;32(1):68. doi: 10.1186/1756-9966-32-68 (PMC3849007; doi:10.1186/1756-9966-32-68)
Supplement: Additional file 1 — Cytotoxicity of 2 and 3 and SPR sensorgrams. [file 1756-9966-32-68-S1.pdf]

Supplemental Figure 1

NCI data for compound **2**

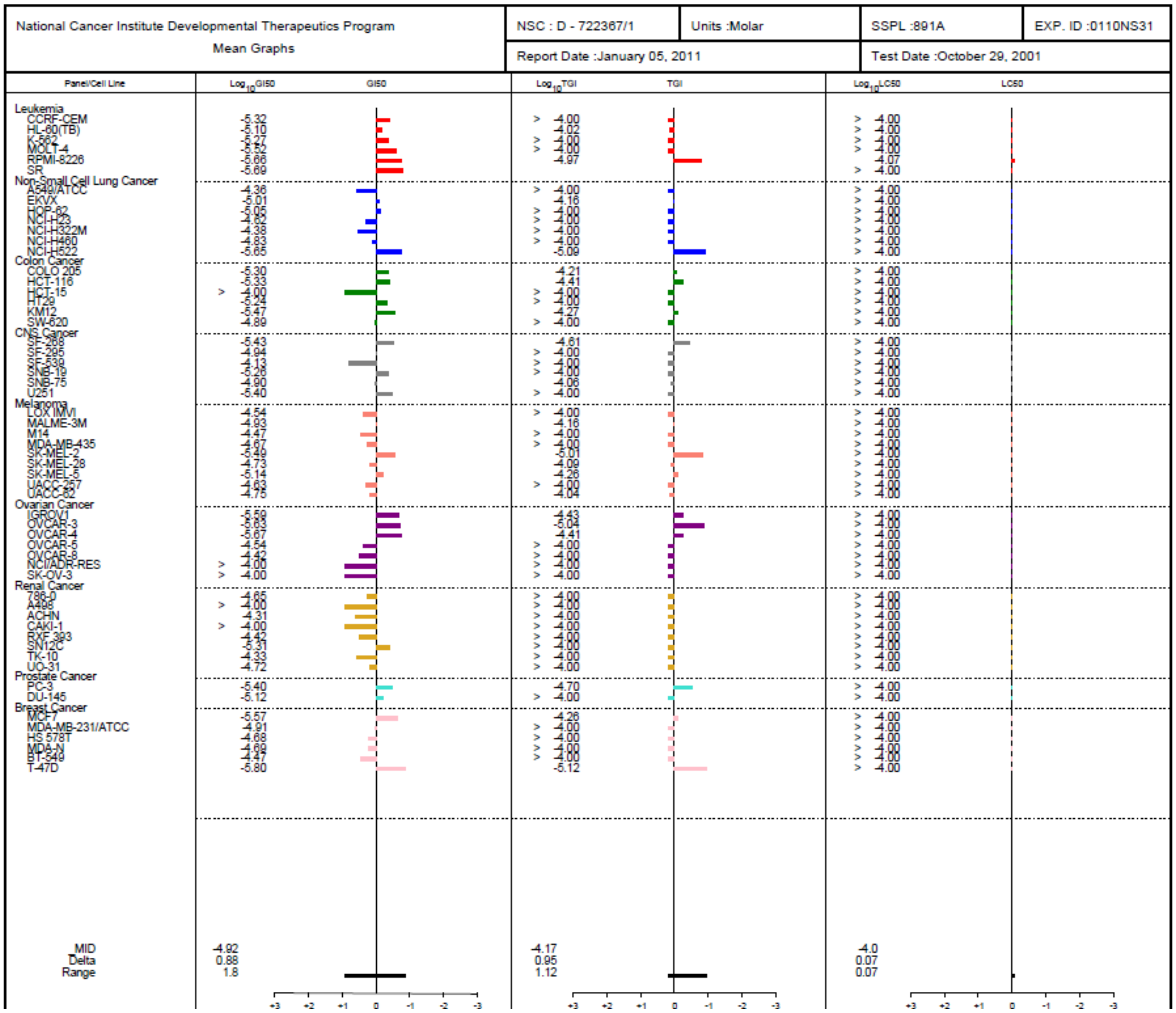

Mean GI_{50} value from the NCI60 panel for compound **2** = 12.02μM

Supplemental figure 2

NCI data for compound **3**

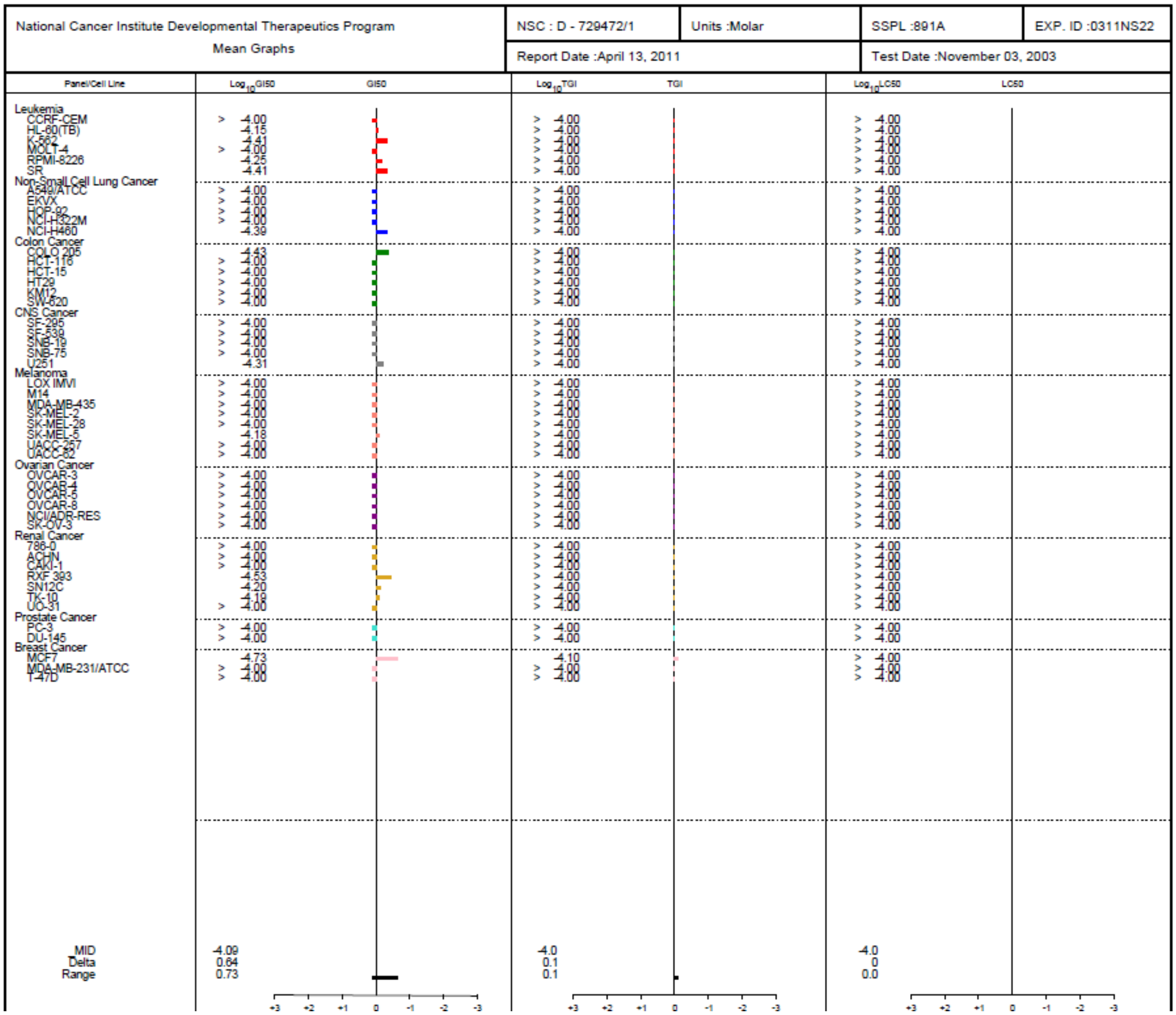

Mean GI_{50} value from the NCI60 panel for compound **3** = 81.28μM

Supplemental Figure 3

a

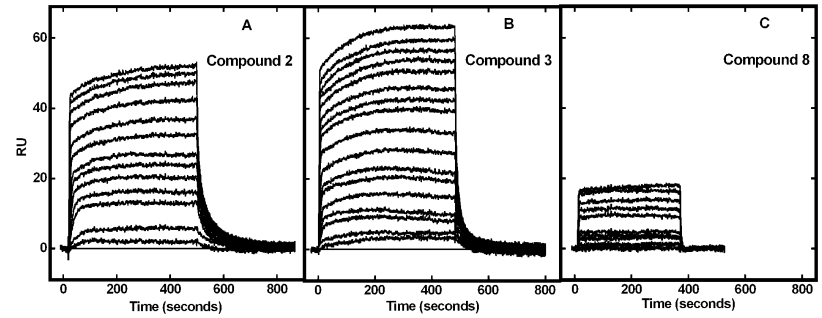

b

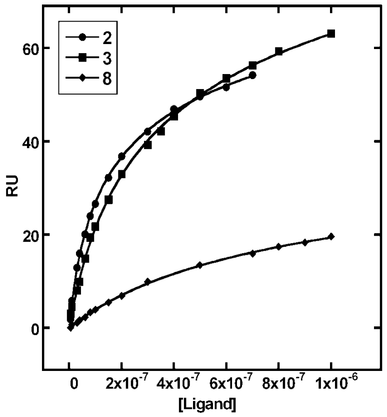

c

Compound	*h*-Tel $Kx10^7 M^{-1}$	Duplex $Kx10^7 M^{-1}$
**1**	0.83	0.05
**2**	1.5	0.04
**3**	0.80	0.07
**8**	0.06	<0.005

d

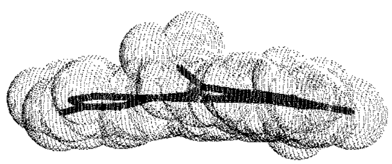

1

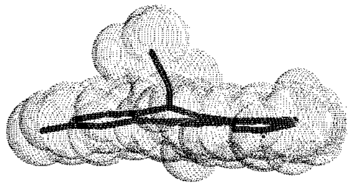

8

a: SPR sensorgrams for the interaction of the compounds **2** (A), **3** (B) and **8** (C) with 5'-biotin labeled human telomeric DNA (*h*-Tel). The individual sensorgrams (colored) represent responses at different ligand concentrations. Increasing ligand concentrations varying from 5 nM up to 1 μM (0.7 μM for compound **2**) were injected over the sensor-chip surface. **b**: SPR equilibrium binding plots of compounds **1**, **2**, **3** and **8** with *h*-Tel DNA. The steady-state response values were fitted as a function of free ligand concentration to a single-site interaction model**.** **c**: Binding affinities. **d**: Representative schematic model of compounds **1** and **8** Illustration is based on solvent accessibility demonstrating that an increase in steric bulk at N-8 corresponds to a decrease in planarity.
